# Integrative Analysis of the Predictive Value of Perilipin Family on Clinical Significance, Prognosis and Immunotherapy of Glioma

**DOI:** 10.3390/biomedicines11041009

**Published:** 2023-03-24

**Authors:** Xuanxuan Li, Kuo Kang, Lin Shen, Liangfang Shen, Yangying Zhou

**Affiliations:** 1 Department of Oncology, Xiangya Hospital, Central South University, Changsha 410008, China; 2 National Clinical Research Center for Geriatric Disorders, Xiangya Hospital, Central South University, Changsha 410008, China; 3 Department of General Surgery, Xiangya Hospital, Central South University, Changsha 410008, China; 4 Hunan Key Laboratory of Precise Diagnosis and Treatment of Gastrointestinal Tumor, Xiangya Hospital, Central South University, Changsha 410008, China

**Keywords:** *PLINs* family, glioma, clinical prognosis, immune microenvironment, biomarkers

## Abstract

Gliomas are common tumors of the central nervous system. The *PLINs* family is widely involved in the regulation of lipid metabolism and has been associated with the development and invasive metastasis of various malignancies. However, the biological role of the *PLINs* family in gliomas is still unclear. TIMER and UALCAN were used to assess *PLIN*s mRNA expression in gliomas. “Survminer” and “Survival” were used to evaluate the connection between *PLINs* expression and glioma patients’ survival. cBioPortal was applied to assess *PLIN*s’ genetic alterations in glioblastoma multiforme (GBM) and low-grade glioma (LGG). The correlation of *PLINs* expression with tumor immune cells was analyzed by TIMER. The expressions of *PLIN1*, *PLIN4*, and *PLIN5* were decreased in GBM compared to normal tissues. However, *PLIN2* and *PLIN3* were significantly increased in GBM. Prognostic analysis showed that LGG patients with high *PLIN1* expression had better overall survival (OS), and high expression of *PLIN2/3/4/5* was associated with unfavorable OS. We further determined that the expression of *PLINs* members in gliomas was strongly related to tumor immune cells and immune checkpoint-associated genes. *PLINS* may be potential biomarkers for regulating the tumor microenvironment and predicting the efficacy of immunotherapy. In addition, we determined that *PLIN1* may affect glioma patients’ therapeutic sensitivity to temozolomide. Our results demonstrated the biological significance and clinical values of *PLINs* in gliomas and provide a basis for future in-depth exploration of the specific mechanisms of each member of *PLINs* in gliomas.

## 1. Introduction

Gliomas are a common group of primary malignant brain tumors with heterogeneous nature and poor prognosis. According to the World Health Organization (WHO) classification of 2021, histologically gliomas can be categorized as astrocytic tumors, oligoastrocytic tumors, oligodendroglia tumors, mixed neuronal-glial tumors, and ependymal tumors. In consideration of molecular genetic factors, gliomas are also divided into astrocytoma, *IDH*-mutant, oligodendroglioma, *IDH*-mutant and 1p/19q-codeleted, glioblastoma, and *IDH*-wildtype [1]. The WHO Grade IV gliomas are also known as glioblastoma multiforme (GBM) [2]. It is estimated that more than 50% of malignant gliomas are glioblastoma multiforme [3]. GBM has the characteristics of a high degree of malignancy, strong invasiveness, and heterogeneity of cells, and the survival time is 12–18 months [4]. Although low-grade gliomas (LGG, WHOI/II) grow slowly, they may evolve into secondary glioblastomas during the long-term progression of the disease [5]. At present, there have been significant advances in the treatment options for gliomas, but the limited treatment efficacy and poor prognosis of gliomas remain a thorny issue for clinical workups. Therefore, more biomarkers need to be explored to further improve the treatment strategy for glioma and improve patient prognosis and survival.

Abnormalities in tumor lipid metabolism have gradually attracted the attention of researchers. Abnormal accumulation of lipids in tumor cells usually occurs in the form of lipid droplets (LDs) [6]. Lipid droplets are essential organelles for lipid storage in cells, and play an important role in lipid storage and energy homeostasis during normal body physiological activities [7]. The abnormal accumulation of LDs may promote tumorigenesis by affecting the tumor microenvironment, immune response, synthesis of inflammatory mediators, and cell signaling pathways [8,9].

The surface of lipid droplets contains a variety of proteins involved in lipid metabolism, membrane transport, and protein degradation [10]. In mammals, the most characteristic surface protein belongs to the PAT family, also known as the perilipin protein. Perilipin proteins are major structural proteins located on the surface of lipid droplets and are involved in a variety of biological activities such as intracellular LD formation and degradation, signaling, cytoskeleton formation, and regulation of lipid metabolism [11,12]. In mammals, *PLINs* are composed of *PLIN1* (perilipin-1), *PLIN2* (also called adipose differentiation-related protein, adipophilin, and perilipin-2), *PLIN3* (perilipin-3, TIP47, placental protein 17), *PLIN4* (perilipin-4, adipocyte protein S3-12, KIAA1881), and *PLIN5* (perilipin-5, LSDP5, lipid storage droplet protein 5).

*PLIN2* and *PLIN3* are widely distributed in various tissues and cell types, *PLIN1* and *PLIN4* are present in adipocytes, and *PLIN5* is usually highly expressed in skeletal muscle, liver, and brown fat [13,14]. The *PLINs* family has been studied in metabolism-related diseases such as hepatic steatosis, cardiovascular diseases, and diabetes [15,16,17]. Perilipins are considered a biomarker of sebaceous gland epithelial cells and myoepithelial cell carcinogenesis in parotid cancer [18]. It has also been demonstrated that *PLIN1*, *PLIN2*, and *PLIN3* are co-expressed in hepatocellular carcinoma [19]. *PLIN4* mutations have been identified in gastric and lung cancers and are associated with poor prognosis [20]. Zhang et al. [21] showed that *PLIN3* is highly expressed in breast cancer while *PLIN1/2/4/5* show low expression in breast cancer and *PLIN1* and *PLIN2* are associated with prognosis in breast cancer patients. However, the clinical significance and potential function of the *PLINs* family in glioma are poorly understood.

Therefore, in this study, we systematically explored the mRNA and protein expression levels of the *PLINs* family in gliomas and their relationship with clinical implications. We revealed that the expression of *PLINs* in glioma is closely related to clinicopathological characteristics, survival prognosis, tumor immune microenvironment, and sensitivity to targeted therapeutic drugs. Members of *PLINs* may serve as prospective therapeutic targets for glioma and improve patients’ clinical outcomes.

## 2. Materials and Methods

### 2.1. Study Design and Data Collection

In this study, clinical data and gene expression data of all glioma patients (LGG and GBM) were derived from The Cancer Genome Atlas (TCGA) and the Chinese Glioma Genome Atlas (CGGA) databases. The gene expression microarray data of 1018 glioma samples were downloaded from the CGGA dataset. RNA-seq data from 706 glioma samples were obtained from the TCGA database (TCGA-LGG and TCGA-GBM). We utilized Verhaak’s gene expression-based molecular classification of GBM, which includes the proneural, neural, classical, and mesenchymal subtypes [22]. All histological subtypes of gliomas were included in this study. The clinicopathological characteristics of patients from the different databases are summarized in Supplementary Tables S1 and S2.

The inclusion criteria for this study were: (1) histological diagnosis of glioma; (2) available gene expression data; and (3) available survival data. Cases with insufficient or missing overall survival time were excluded.

### 2.2. Expression Levels of PLINs in Pan-Cancer and Gliomas

The Tumor Immune Estimation Resource (TIMER2; http://timer.cistrome.org/ (accessed on 1 December 2022 )) is an interactive web server that includes clinical data for 32 cancer types in the TCGA database, capable of analyzing and visualizing the linkages between tumor immunological and clinical features [23]. We used the ”Gene DE module” to assess the gene expression levels of the *PLIN* family between tumor tissue and matched normal tissue. UALCAN (http://ualcan.path.uab.edu/analysis.html (accessed on 1 December 2022)) is an interactive web portal based on RNA-seq transcriptome data from TCGA datasets. Users can utilize the data from TCGA to analyze the relationship between genes of interest in different cancer types and subgroups and clinical features. We used UALCAN to assess the mRNA expression levels of *PLINs* family members in GBM and normal tissues [24,25]. We downloaded protein expression data of *PLINs* from the Clinical Proteomic Tumor Analysis Consortium (CPTAC) and the Human Protein Atlas project (HPA; https://www.proteinatlas.org (accessed on 1 December 2022)) databases [26,27].

### 2.3. Correlation of PLINs Expression with Survival Prognosis and Clinicopathological Features of Glioma

Gliovis (http://gliovis.bioinfo.cnio.es/ (accessed on 1 December 2022)) is a database for analyzing and visualizing brain tumor expression data. It includes over 50 brain tumor datasets [28]. In this study, we used the CGGA database to analyze the differences in mRNA expression levels between members of different *PLINs* in terms of WHO classification, recurrence status, *IDH* status, and 1p/19q codes of gliomas.

By extracting the survival information of LGG and GBM samples from TCGA database, we analyzed and visualized the relationship between *PLINs* expression and overall survival in LGG and GBM patients using the R packages “Survminer” and “Survival”. Kaplan-Meier survival curves were plotted. Hazard ratios (HRs) and log-rank *p*-values with 95% confidence intervals (CI) were also calculated [29]. And We used Xiantao online tools (https://www.xiantao.love/ (accessed on 1 December 2022)) for mapping.

### 2.4. Genetic Alterations of PLINs Family in Glioma

cBioPortal (https://www.cbioportal.org/ (accessed on 1 December 2022)) is a multidimensional cancer genomic data platform that contains datasets from 10 published cancer studies and can explore whether genetic alterations such as mutations, homologous gene deletions, gene amplifications, increased or decreased mRNA expression, and increased or decreased protein abundance occur in target genes [30,31]. We analyzed the genetic alterations of the *PLINs* family in GBM and LGG through cBioPortal.

### 2.5. Association Analysis of PLINs Expression with Tumor Immune Microenvironment in Glioma

The correlation of *PLINs* expression with the level of immune infiltration in GBM and LGG was investigated through GSCA and TIMER databases. We then used the Sangerbox website to evaluate the relationship between *PLINs* expression and ESTIMATScore, StromalScore, and immuneScore in GBM and LGG. According to the previous study, we selected 58 of two types of immune checkpoint markers [32,33]. The expression of these checkpoints in different cancers was extracted from the TCGA database, and the normal samples were filtered. The relationship between the expression of immune checkpoint-related genes and *PLINs* in gliomas was calculated by Pearson correlation analysis.

### 2.6. Correlation and Functional Enrichment Analysis of PLINs Family

GeneMANIA (http://genemania.org/ (accessed on 1 December 2022)) can be used for gene function prediction and interactions. By entering the *PLINs* family in the GeneMANIA search box, the website automatically generates predicted *PLINs* interactions and builds interaction networks. Click on “Functions” to obtain the functions they are involved in. To further understand the functions of *PLINs* in LGG, we screened 500 genes interacting with the *PLINs* family in LGG using LinkedOmics (http://www.linkedomics.org/ (accessed on 1 December 2022)) [34]. We performed Gene Ontology (GO) enrichment and Kyoto Encyclopedia of Genes and Genomes (KEGG) pathway analysis and visualization using the METASCAPE network (http://metascape.org (accessed on 1 December 2022)) [35].

### 2.7. Drug Sensitivity Analysis

We utilized the genomic and pharmacogenomic data from the Gene Set Cancer Analysis (GSCA, http://bioinfo.life.hust.edu.cn/GSCA/#/ (accessed on 1 December 2022)) database, which contains more than 10,000 genomic datasets of 33 TCGA cancer types, as well as details on small-molecule drugs from the Genomics of Drug Sensitivity in Cancer (GDSC) and the Cancer Therapeutics Response Portal (CTRP). We used the GSCA database in the “Drug sensitive” module of the website to analyze the drug sensitivity associated with the *PLINs* family. It mainly analyzed the correlation between gene mRNA expression levels and drug IC50, with a negative correlation indicating that high gene expression is more sensitive to drugs.

The CellMiner database (http://discover.nci.nih.gov/cellminer/ (accessed on 18 February 2023)) provides access to data on 22,379 identified genes in NCI-60 cell lines and 20,503 analyzed compounds [36]. We used CellMiner to access the NCI-60 compound activity data to analyze the drug sensitivity of *PLINs* in glioma. The drug without FDA approval was excluded. A positive correlation indicates that drug sensitivity is positively correlated with PLINs expression, while a negative correlation represents the higher expression of PLINs may be related to less drug sensitivity.

## 3. Results

### 3.1. Expression Levels of PLINs in Pan-Cancer and Gliomas

**Figure 1:**
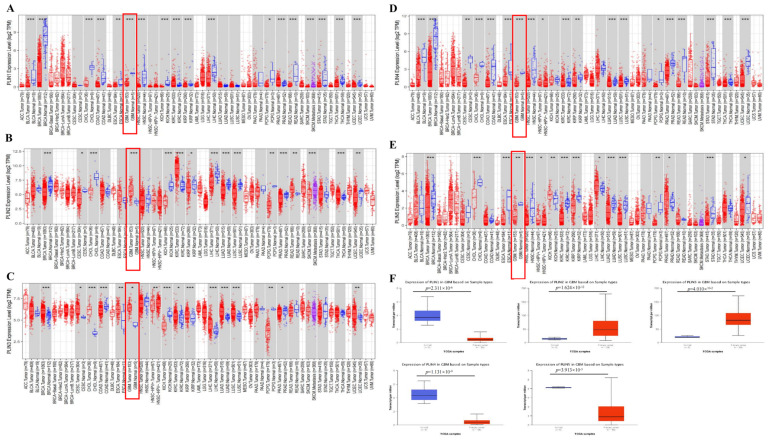
*PLINs* family members’ mRNA expression in pan-cancer. (**A**–**E**) Expression levels of perilipin members (*PLINs)* gene in pan-cancer according to TIMER2.0 database. * *p* < 0.05; ** *p* < 0.01; *** *p* < 0.001. (**F**) Expression levels of *PLINs* gene in tumor and normal tissues based on UALCAN database.

Firstly, we analyzed the expression of each *PLINs* family member in pan-cancer using the TIMER 2.0 database to clarify their differential expression in human tumors. The results indicated that each *PLINs* family showed a decreased expression pattern in most cancer types (Figure 1A–E; red represents tumor tissue and blue represents normal tissue). Likewise, according to the TIMER 2.0 database, mRNA expressions of *PLIN1*, *PLIN4*, and *PLIN5* were decreased in GBM compared to normal tissues. However, *PLIN2* and *PLIN3* were obviously increased in GBM compared to normal tissues. We further explored the transcript levels of *PLINs* in GBM using the UALCAN database and obtained similar results (Figure 1F).

**Figure 2:**
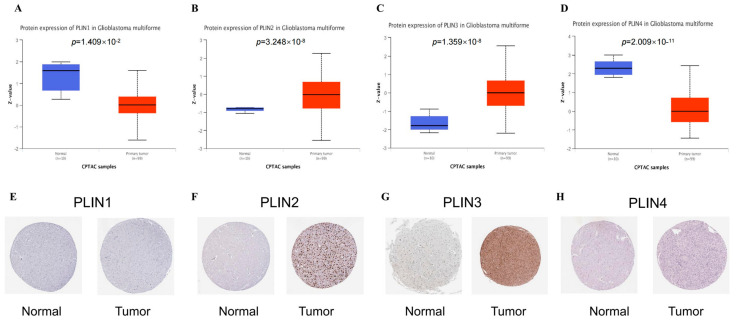
The levels of protein expression of *PLINs* in GBM. (**A**–**D**) In the CPTAC database, *PLIN2/PLIN3* had increased protein levels in GBM and *PLIN1/PLIN4* had decreased protein levels in GBM compared to normal tissue. (**E**–**H**) Protein expression of *PLINs* from the HPA database.

Using the CPTAC and HPA databases, we further analyzed the protein expression of the PLINs. In contrast to normal tissues, *PLIN2* and *PLIN3* were found to be overexpressed, which was compatible with the mRNA expression data. We also revealed that PLIN1 and PLIN4 were downregulated in GBM tissues from the CPTAC database, while we have not observed obvious differences in PLIN1 and PLIN4 expression in the HPA dataset. This needs further investigation (Figure 2).

### 3.2. The Correlation between PLINs Family and Clinicopathological Features in Glioma Patients

After clarifying the expression of each member of *PLINs* in gliomas, we further explored the relationship between the gene members of *PLINs* and different pathological features of gliomas using the GlioVis database. We selected RNA-Seq data from the CGGA database of 1013 patients from GlioVis. We compared the expression differences of *PLINs* in different subgroups stratified by WHO classifications, *IDH* mutation, 1p/19q coding, and recurrence status.

**Figure 3:**
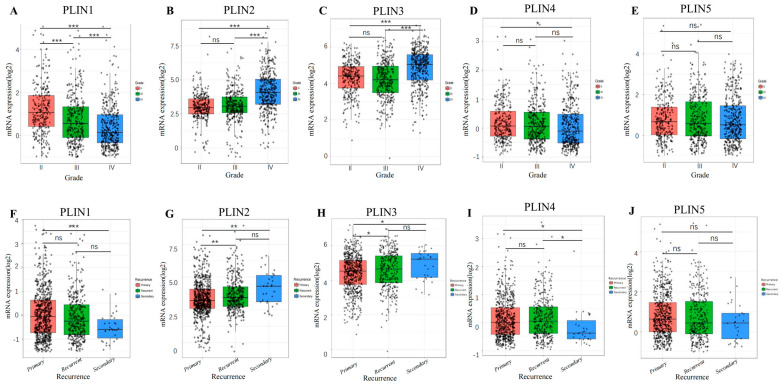
Correlation analysis of *PLINs* and clinical features of glioma. (**A**−**E**) The mRNA expression levels of *PLIN1-5* in different glioma World Health Organization classifications (* *p* < 0.05; ** *p* < 0.01; *** *p* < 0.001, ns: no significance) (**F**−**J**) Comparison of mRNA expression levels of *PLIN1*−*5* in primary, secondary, and recurrent glioma.

Firstly, we analyzed the relationship between *PLINs* and the WHO grade of glioma. We determined that the mRNAs of each family member of *PLINs* were correlated with glioma tumor grade (Figure 3A–E). Among them, the mRNA expression level of *PLIN1* gradually decreased with the increase in tumor grade, while *PLIN2* showcased an increasing trend. However, there was no significant difference in the mRNA expression of *PLIN5*. mRNA levels of *PLIN2* and *PLIN3* were not statistically different between grade II and grade III, and the highest mRNA levels of both were expressed in grade IV glioma tissues. The results suggested that *PLINs* were closely related to the malignancy of gliomas.

We further investigated whether there was a correlation between *PLIN1*/2/3/4/5 expression and the recurrence status of glioma (Figure 3F–J). The results demonstrated that the mRNA levels of *PLIN1*/*PLIN4*/*PLIN5* were not correlated with tumor recurrence status, while *PLIN2* and *PLIN3* were correlated with tumor recurrence status. Furthermore, we observed that the expression levels of *PLIN2* and *PLIN3* were higher in the recurrent tissues than in the primary tumors, indicating that they may play essential roles in the development and recurrence of gliomas.

**Figure 4:**
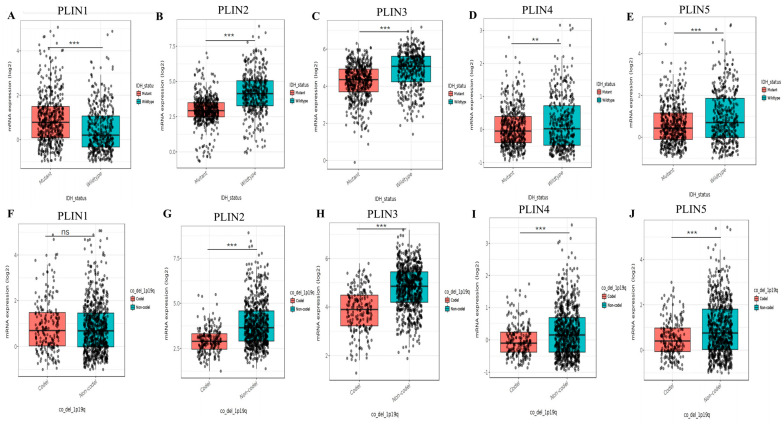
Correlation analysis of *PLINs* with molecular pathological features of glioma. (**A**–**E**) Differential mRNA expression levels of *PLIN1*−*5* in glioma IDH mutant and wild type (** *p* < 0.01; *** *p* < 0.001, ns: no significance). (**F**–**J**) Differential mRNA expression levels of *PLIN1*−*5* in glioma 1p/19q coding and non-coding (** *p* < 0.01; *** *p* < 0.001, ns: no significance).

We also explored the relationship between *PLINs* mRNA expression levels and *IDH* mutation status (Figure 4A–E). We explored that *PLIN2/3/4/5* were highly expressed in all *IDH* wild-type gliomas in the CGGA dataset. 1p19q deletion is a pathological subtype of glioma. We further analyzed the relationship between *PLINs* mRNA expression and 1p19q deletion/non-deletion status, and in the CGGA cohort, *PLIN2-5* expression levels were increased in the 1p19q non-deletion status (Figure 4F–J). These results suggested that *PLINs* can be used as molecular biomarkers for predicting glioma subtypes in gliomas.

### 3.3. Prognostic Value of PLINs mRNA Expression in Glioma Patients

**Figure 5:**
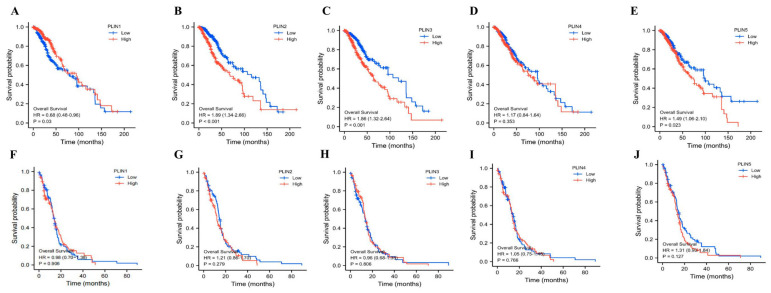
Prognostic value of *PLINs* family in gliomas. (**A**–**E**) Overall survival analysis of *PLIN1-5* mRNA high and low expression in LGG. (**F**–**J**) Overall survival analysis of *PLIN1-5* mRNA high and low expression in GBM.

From the above results, we determined that *PLINs* family members are specifically expressed in gliomas, and most are associated with malignant clinicopathological features in glioma patients. Therefore, we further explored the impact of the *PLINs* family on survival in both LGG and GBM in TCGA-LGG and TCGA-GBM cohorts. As illustrated in Figure 5, the results showed that in the TCGA-LGG cohort, mRNA of *PLINs* was apparently associated with the prognosis of LGG patients. The patients with high *PLIN1* expression had better overall survival (OS). However, the high expression of *PLIN2*/3/5 was associated with an inferior OS, while no statistical survival difference was observed in GBM patients. In addition, we evaluated the prognostic impact of the *PLINs* family stratified by *IDH* genotyping and 1p/19q (Supplementary Figure S1). Kaplan–Meier curves demonstrated that in the *IDH* subgroup, high expression of *PLINs* was not significantly correlated with patients’ OS. In the 1p/19q non-codel subgroup, patients with high *PLIN1* expression had a superior median survival time than those with low *PLIN1* expression, whereas patients with high *PLIN2*, *PLIN3,* and *PLIN4* expression had a worse OS than those with low expression.

### 3.4. Genetic Alterations of PLINs in Glioma

**Figure 6:**
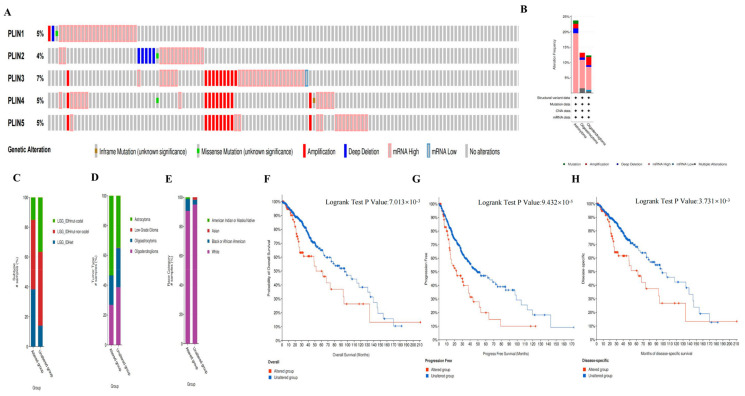
Gene alterations in the *PLINs* family in LGG. (**A**–**B**) Frequency and type of genetic alterations of *PLINs* in LGG. (**C**) Patients with *IDH* wild type in LGG were more likely to have family alterations in *PLINs*. (**D**) Astrocytoma was more likely to have *PLINs* family gene mutations. (**E**) Blacks or African American LGG patients were more likely to have family alterations in *PLINs.* (**F**–**H**) Prognostic analysis of genetically altered and non-altered groups of *PLINs* families in LGG. (+: contains the included data).

In the cBioPortal database, we explored the genetic alterations of *PLINs* family members in LGG and GBM (Figure 6, Supplementary Figure S2). We found that the overall mutation frequency of *PLINs* in LGG was 17%, with *PLIN3* having the highest alteration frequency (7%), while *PLIN1*, *PLIN4,* and *PLIN5* were all altered at about 5%, and *PLIN2* was altered at 4% (Figure 6A). We determined that the most common gene alteration patterns in LGG are mRNA high, amplification, and deep deletion (Figure 6B). While *PLINs* are more conservative in GBM compared to LGG with a mutation frequency of 5%, the *PLIN1*-5 of GBM had a mutation frequency of 0.5%, 1.6%, 1.6%, 2.6%, and 1.9%, respectively (Supplementary Figure S2).

Next, we analyzed the relationship between genetic alterations in *PLINs* and LGG-related clinical characteristics. Our results revealed that in patients with pathological types of LGG, *IDH*-WT was more frequently explored in the group with *PLINs* mutations (Figure 6C). Astrocytoma was more likely to have *PLINs* family gene mutations (Figure 6D). Whites were more likely to have familial mutations in *PLINs* compared to Blacks or African Americans, whereas, in Asian ethnic groups, most Asians do not have alterations in *PLINs* (Figure 6E).

In addition, we analyzed the correlation between genetic alterations in *PLINs* and survival outcomes in LGG and GBM patients. Our results demonstrated that in LGG, genetic alterations in *PLINs* were associated with inferior OS, progression-free survival (PFS), and disease-specific survival (DSS) between the two groups of patients (Figure 6F–H). In contrast, in GBM patients, there was no statistically significant difference in the correlation between genetic alterations of *PLINs* and the prognosis of GBM patients (Supplementary Figure S2).

### 3.5. Correlations between the Immune Microenvironment of Glioma and the Expression of PLINs

**Figure 7:**
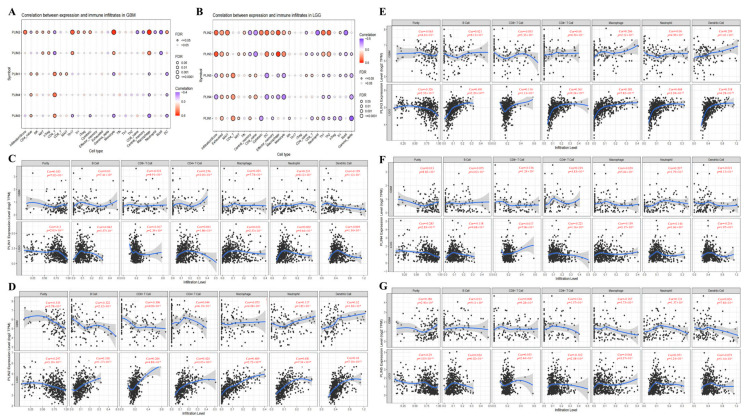
Correlation analysis of *PLINs* family and immune microenvironment of glioma tumors. (**A**,**B**) The overall relationship between *PLINs* and tumor immune cells was analyzed through the GSCA online website. (**C**–**G**) Correlation analysis of *PLIN1*−*5* with tumor immune cells in LGG and GBM.

Immune cell infiltration plays an essential role in tumor progression and recurrence. We first analyzed the overall relationship between *PLINs* and tumor immune cells using the GSCA database. Next, the correlation between immune cell infiltration and *PLINs* expression was explored in depth using the TIMER database. In LGG, there was a consistent positive correlation between the expression of *PLINs* and exhausted T cells, mucosal-associated invariant T cells (MAIT), and CD4^+^ T cells. In contrast, a persistent negative correlation was observed in B cells and gamma delta T cells, but a constant negative correlation between *PLINs* expression and B cells was only observed in GBM (Figure 7A,B).

The data of six immune infiltrating cells in LGG and GBM were also retrieved from the TIMER database. The results revealed that *PLIN1* was negatively correlated with CD8^+^ T cells in LGG, and positively correlated with CD4^+^ T cells and neutrophils in GBM (Figure 7C). *PLIN2* was positively associated with B cells, CD8^+^ T cells, CD4^+^ T cells, macrophages, neutrophils, and dendritic cells in LGG. In GBM, *PLIN2* exhibited a positive correlation with neutrophils and dendritic cells, and a negative correlation with B cells and CD8^+^ T cells (Figure 7D). *PLIN3* has a positive correlation in LGG with B cells, CD8^+^ T cells, CD4^+^ T cells, and macrophages (Figure 7E). *PLIN4* was negatively correlated with B cells, CD4^+^ T cells, macrophages, neutrophils, and dendritic cells in LGG and positively associated with CD8^+^ T cells and dendritic cells in GBM (Figure 7F). *PLIN5* was negatively correlated with CD4^+^ T cells in LGG, while it was not statistically correlated with immune cells in GBM (Figure 7G).

We subsequently evaluated the stromal, immune, and ESTIMATE scores for patients in the TCGA using the ESTIMATE algorithm to investigate whether the expression of *PLINs* members correlates with the degree of immune invasion and the level of infiltrating stromal and immune cells in gliomas (Supplementary Figures S3 and S4). The results revealed that in LGG, the expressions of *PLIN2* and *PLIN3* were positively associated with the immune score, stromal score, and ESTIMATE score, while *PLIN4* was negatively associated with the immune score and ESTIMATE score in LGG (Supplementary Figure S3). In contrast, in GBM, we observed that *PLIN2* and *PLIN3* were positively related to the immune, stromal, and ESTIMATE scores. *PLIN5* was negatively correlated with the immune, stromal, and ESTIMATE scores, while *PLIN1* and *PLIN4* were not significantly associated with these three scores (Supplementary Figure S4).

### 3.6. Correlation of PLINs with Immune Checkpoints and Drug Sensitivity in Gliomas

**Figure 8:**
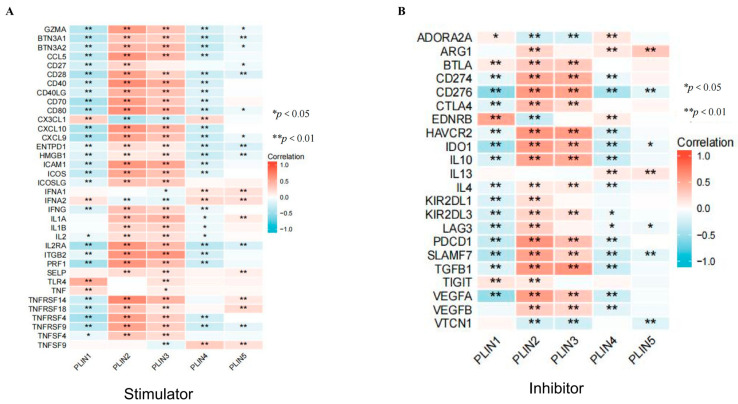
Correlation of *PLINs* with immune checkpoint genes in gliomas. (**A**) Immunostimulatory factors. (**B**) Immunosuppressive factors. (* *p* < 0.05; ** *p* < 0.01).

**Figure 9:**
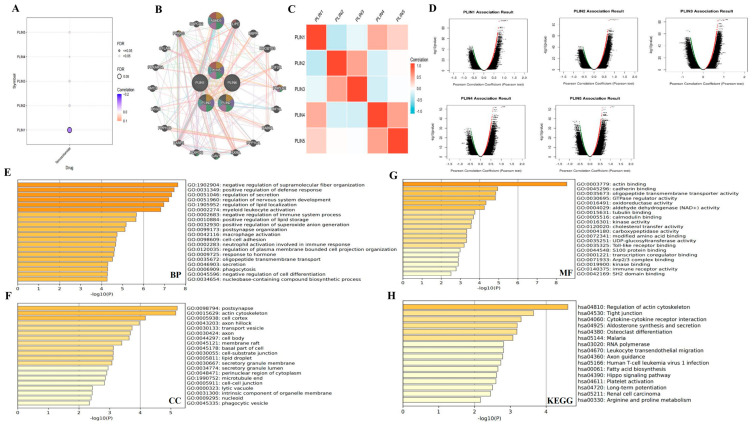
Analysis of drug sensitivity-associated genes and biological functions of *PLINs* family. (**A**) Drug sensitivity analysis of *PLINs* family. (**B**) The *PLINs* gene interaction network and related functions were obtained by GenMANIA analysis. (**C**) Heat map of the correlation between the members of the *PLINs* family. (**D**) Volcano maps of *PLINs* family members with co-expressed genes in LGG. (**E**–**H**) GO enrichment of *PLINs* family members in LGG and KEGG pathway analysis. GO: Gene ontology.

Immunotherapy alters the paradigm of systemic cancer treatment. We investigated the relationship between glioma *PLINs* expression and immunological checkpoints. The results demonstrate that members of *PLINs* have predictive efficacy for immunotherapy aimed at targeting immune checkpoint genes (Figure 8). We explored whether *PLIN2/PLIN3* were positively associated with a large number of immune checkpoint inhibitors, such as *CTLA4*, *CD274* (PD-L1), and *HAVCR2*, etc. Except for *ARG1* and *EDNRB*, *PLIN1/4/5* were negatively correlated with a large number of immune checkpoints (Figure 8). Moreover, we explored the relationships between *PLINs* and the sensitivity of conventional chemotherapeutic medications and targeted therapeutic agents for gliomas. We performed a sensitivity analysis of *PLINs* in the GSCALiteOnline Tool, and the results revealed that *PLIN1* expression was negatively associated with the sensitivity of the targeted drug temozolomide (Figure 9A). We further used the Cellminer database to assess the correlation between the expression of *PLINs* and anti-tumor drug sensitivity in glioma (Supplementary Figure S5). We revealed that *PLIN1* expression was negatively correlated with drug responsiveness in patients treated with trametinib (Cor = −0.279, *p* = 0.033), AEE-788 (Cor = −0.273, *p* = 0.036), and adavosertib (Cor = −0.290, *p* = 0.026). *PLIN2* expression was positively associated with dabrafenib (Cor = 0.379, *p* = 0.003) and selumetinib (Cor = 0.323, *p* = 0.013). *PLIN3* was positively related to telatinib (Cor = 0.352, *p* = 0.006). *PLIN4* was negatively associated with the drug responsiveness of trametinib (Cor = −0.362, *p* = 0.005) patients and *PLIN5* was positively related to linsitinib (Cor = 0.340, *p* = 0.008).

### 3.7. Interaction Networks and Gene Enrichment Analysis of PLINs Family Genes in Glioma

To investigate the interacting genes and potential functions of PLNs, we first mined the gene interaction network of *PLINs* family members in glioma using the Gene MANIA database (Figure 9B). The network revealed 20 genes with close relational–functional relationships between *PLINs* members, including *ABHD5, LIPE, M6PR, RHOBTB3, IGF2R, PNPLA2, HIF1A, RAB9A, PPP1CA, RXRA, CAV1, ADIPOQ, FABP4, PPP1CB, DSG2, SMC3, PRKAR2A, CFLAR, PPARG,* and *SAPCD2*. These genes are mutually expressed, and have a functional relationship with regulation of triglyceride sequestering, lipid localization, lipid storage, and the acylglycerol catabolic process, which is consistent with the biological role of *PLINs*. We further determined that 77.64% of the *PLINs* showed physical interaction with each other and these 20 genes. In addition, there were correlations in mRNA expressions between the different members of the *PLINs* family members in glioma patients (Figure 9C).

Next, we evaluated the correlations of *PLINs* family members with 500 co-expressed genes in LGG using LinkedOmics (Figure 9D, Supplementary Figure S6). GO enrichment and KEGG pathway analysis were also performed in MetaScape based on the 500 associated genes. The GO functional annotations included biological process (BP), cellular composition (CC), and molecular function (MF). As depicted in Figure 9E, the pathways with the highest enrichment of BP were mainly involved in the regulation of supramolecular fiber organization, defense response, secretion, nervous system development, lipid localization, and myeloid leukocyte activation. In CC, co-expressed genes were primarily localized in post-synapse and actin cytoskeleton (Figure 9F). The enriched terms for MF comprised actin binding, cadherin binding, and oligopeptide transmembrane transporter activity (Figure 9G). In addition, the KEGG pathway analysis demonstrated that related genes have mainly participated in the regulation of the actin cytoskeleton, tight junction, and cytokine–cytokine receptor interaction (Figure 9H).

## 4. Discussion

In this study, we comprehensively analyzed the expression of *PLINs* family members in glioma patients, their genetic alterations, and their relationship with immune cell infiltration. We revealed that mRNA expression levels of *PLINs* family members were all up-regulated in gliomas compared to normal brain tissue, and were correlated with WHO classification, recurrence status, *IDH* typing, and 1p/19q coding type. Furthermore, survival analysis showed that *PLIN2*/3/5 were influential factors for poor LGG prognosis, while *PLIN1* was associated with better OS, which suggested that the expression of some members of *PLINs* may be involved in the malignant behavior of gliomas and could be vital biomarkers for predicting LGG prognosis.

The *PLINs* family has five members (*PLIN1-5*) that play essential roles in lipid metabolism and carcinogenesis. There are some differences and commonalities among the *PLINs* members because they have variations in tissue distribution, molecular size, and affinity for LD binding. In recent years, more has been learned about the metabolic regulatory role of the *PLINs* family in tumors. Many experimental studies have found that the *PLINs* family is expressed at increased levels in tumor tissues such as breast cancer, hepatocellular carcinoma, and clear cell renal carcinoma and affects patients’ prognosis [19,37,38,39]. However, the expression of the *PLINs* family in glioma and its prognostic significance remains unknown. In this study, we determined that *PLINs* were associated with patients’ clinical features, survival outcomes, as well as tumor immune microenvironment and drug therapeutic efficacy and may become a prospective biomarker for glioma.

*PLIN1* is mainly an adipocyte protein; the encoded protein can protect stored lipid droplets in adipocytes from being broken down by lipase and has the effect of inhibiting lipolysis [40]. Zhang et al. [21] found that *PLIN1* was significantly downregulated in breast cancer and that high *PLIN1* expression was significantly associated with better OS in breast cancer and ER^+^ subtypes. Zhou et al. [39] observed a similar result that *PLIN1* expression was reduced in breast cancer and that low *PLIN1* expression was a predictor of poor prognosis in breast cancer. Consistent with previous research on breast cancer, the present study demonstrated that *PLIN1* expression was downregulated in GBM, that the expression level fell gradually with increasing WHO classification, and that low *PLIN1* expression was related to an unfavorable outcome in patients with LGG. The mutational status of *IDH* is also an important prognostic factor for glioma patients. Patients with *IDH* wild-type and 1p/19qnon-coding gliomas usually have a poor prognosis and lower treatment sensitivity. Our findings revealed that *PLIN1* was expressed at higher levels in *IDH* mutant compared to the wild type, while there was no significant correlation with 1p/19q. Therefore, *PLIN1* may enhance the malignant progression of gliomas and serve as a promising biomarker for predicting clinical prognosis and drug therapy.

*PLIN2* and *PLIN3* were commonly expressed in tissues, and both share similar amino acid sequences [41]. High expression of *PLIN2* was found to be an unfavorable factor for OS in breast cancer and was associated with aggressive tumor behavior [37]. A previous study reported increased *PLIN2* expression under hypoxic conditions, which in turn promoted increased lipid storage and utilization in tumor cells, which provided favorable conditions for invasive infiltration of cancer cells [42]. Zhang et al. [43] found in lung adenocarcinoma that elevated *PLIN2* was associated with inferior prognosis in lung adenocarcinoma. Elevated levels of *PLIN3* expression were observed in patients with cervical cancer, decreased after treatment, and elevated *PLIN3* was found again at recurrence [44]. Moreover, Zhou et al. demonstrated that the *ACSS3/PLIN3* signaling pathway can boost endoplasmic reticulum stress, which in turn inhibited the advancement of pancreatic cancer [45]. Our findings suggested that *PLIN2* and *PLIN3* expression levels were elevated in glioma. In addition, *IDH* mutation status and 1p/19q co-deletion status have been considered as crucial indicators for the malignant classification and prognosis prediction of glioma [46]. Most glioma patients with *IDH* mutation and 1p19q co-deletion status have a relatively favorable prognosis, which is a vital indicator for stratification in clinical trials [47]. Expression levels of *PLINs* were analyzed in 1p19q non-codel and *IDH* wild-type subtypes, and *PLIN2/3* expression was found to be increased in 1p19q non-codel and IDH wild-type subtypes. Survival analysis showed that *PLIN2* and *PLIN3* expression did not correlate significantly with the survival prognosis of GBM patients, but was associated with shorter survival in LGG patients, and that *PLIN2/PLIN3* overexpression was correlated with the malignant phenotype of gliomas. Therefore, the high expression of *PLIN2* and *PLIN3* in glioma might be related to the malignant progression of glioma and this needs further exploration.

*PLIN4* is mainly explored in white adipose tissues (WAT) and skeletal muscle and it may play a critical role in adipose differentiation [48]. Zhang et al. [49] reported that *PLIN4* expression levels in liposarcoma were similar to *PLIN1*, whereas *PLIN4* expression has not been found in non-liposarcoma, and *PLIN4* can be used as an adjunctive diagnostic indicator for liposarcoma. In the present study, *PLIN4* expression levels were decreased in gliomas, but no statistically significant correlation was found between clinicopathology and survival prognosis; this needs further investigation.

*PLIN5* is mainly identified in oxidative tissues and is involved in the regulation of fatty acid storage and mitochondrial oxidation and is a key factor in the regulation of LDs’ contact with mitochondria [50]. It is highly expressed in skeletal muscle, liver, brown adipose tissue, and adrenal tissue. Asimakopoulou’s study revealed that high *PLIN5* expression was found in hepatocellular carcinoma and could be used as a biomarker for its diagnosis and treatment [51]. The presence of *PLIN5* has also been reported in liposarcomas and rhabdomyosarcomas [52]. *PLIN5* promoted lipid droplet binding to mitochondria, which promoted oxidative phosphorylation, which was more needed by innate immune cells to reduce oxidative phosphorylation when immune activity occurs in the organism. Ye et al. [53] showed that *PLIN5* was associated with longer survival in lung adenocarcinoma. Our study demonstrated that *PLIN5* had low expression in glioma patients and was associated with better survival. It may be that reduced *PLIN5* is promoting lipid droplet and mitochondrial uncoupling thereby enhancing the body’s immune lethality to the tumor.

The tumor microenvironment is a complex environment involving tumor cells, immune cells (B cells, T cells, NK cells, etc.), stromal cells (lymphatic endothelial cells and tumor-associated fibroblasts, etc.), and multiple molecules. The infiltration of immune cells in the tumor microenvironment can affect tumor progression and immunotherapy efficacy [54]. Previous studies have demonstrated that in some cancer types, the infiltration of lymphocytes, especially CD4^+^ T cells and CD8^+^ T cells, was associated with the activation of immune responses and resulted in favorable prognosis. Meanwhile, the tumor lesion infiltration of large numbers of tumor-associated neutrophils may induce inflammation and stimulate tumor progression and metastasis, thus inhibiting anti-tumor T-cell responses [55,56]. Macrophages are multifunctional immune cells that have the function of phagocytosis and digestion of foreign substances. They play an active role in the process from early carcinogenesis to tumor progression [57,58]. The macrophages that are recruited to the tumor microenvironment predominantly play pro-tumorigenic roles and are referred to as tumor-associated macrophages (TAMs) [44]. It was reported that TAMs in TME were activated by suppressive cytokines and chemokines produced by tumor cells. These TAMs can mediate phagocytosis of cancer cells and killing of tumor cytotoxicity, and they interact effectively with the innate and adaptive immune system in a bidirectional manner [59]. In addition, TAMs support tumor growth and metastasis through multiple modalities in angiogenesis, extracellular matrix remodeling, cancer cell proliferation, metastasis, and establishment of immunosuppressive TME and checkpoint blockade of immunotherapy resistance [60]. In this study, we explored the possibility that some *PLINs* may have a specific function in the immunological microenvironments of gliomas by presenting a positive correlation with CD4^+^T cells, CD8^+^T cells, and macrophage infiltration. Since immune responses are complicated and individualized, more experiments are needed to explore the role of *PLINs* in glioma.

We also explored the correlation between *PLINs* and glioma-related targeted drugs and small molecule compounds through the GSCALiteOnline and Cellminer database, and we revealed that *PLIN1* had a significant negative correlation with temozolomide, a drug commonly used in glioma. This suggested that *PLIN1* can be used as a biomarker to predict the sensitivity of glioma patients to temozolomide treatment. Using the Cellminer database, we further determined that *PLINs* expression was correlated with various other drugs’ sensitivities, such as trametinib, dabrafenib, and selumetinib. Of course, future pharmaceutical-related experiments and clinical trials are necessary to better interpret the role of *PLINs* in cancer therapy.

To deeply explore the biological functions and pathways of *PLINs* in glioma, we performed GO and KEGG analyses. GO enrichment results showed that important biological processes of *PLIN* members were involved in the regulation of lipid localization, which was consistent with the previous study. In addition, we demonstrated that the biological processes of *PLIN* members were involved in the regulation of nervous system development and the negative regulation of immune system processes. These analyses suggested that the biological functions of *PLINs* may be relevant to the regulation of neurological development and the immune microenvironment of gliomas.

This study does have certain shortcomings that need to be addressed. First, the vast majority of the data utilized in this investigation was retrieved from publicly accessible sources, and their information bias. Second, to further confirm the clinical application of *PLINs*, a clinical cohort that includes multiple centers and a larger sample size is required. In addition, since the study is preliminary and observational, future in vivo and in vitro validation are required to further investigate the potential role of *PLINs* and possible molecular mechanisms of *PLINs* members in glioma.

## 5. Conclusions

In summary, the expressions of the *PLINs* family were comprehensively analyzed for the first time in glioma. The expressions of *PLINs* were associated with clinical features, patients’ survival outcomes, tumor immune microenvironment, and therapeutic efficacy. Therefore, *PLINs* can be considered as effective and potential biomarkers for glioma patients and provide a fundamental basis for immunotherapeutic targets and precise and individualized therapy exploration for glioma.

## Data Availability

The datasets presented in this study can be found in online repositories. The names of the repository/repositories and accession number(s) can be found in the article/Supplementary Materials.

## Supplementary Materials

The following supporting information can be downloaded at: https://www.mdpi.com/article/10.3390/biomedicines11041009/s1, Figure S1: Prognostic analysis of PLINs family in different subgroups of LGG; Figure S2: Genetic alterations of the PLINs family in GBM; Figure S3: Immune infiltration score of PLINs family in LGG; Figure S4: Immune infiltration score of PLINs family in GBM; Figure S5: Correlations between PLINs expression and drug sensitivity from Cellminer database; Figure S6: Heat map of PLINs family gene-related genes in LGG patients. Table S1: Clinicopathological characteristics of patients from TCGA cohort; Table S2: Clinicopathological characteristics of patients from the CGGA cohort.

## Abbreviations

BP	Biological processes
CC	Cellular components
CI	Confidence intervals
CTRP	Cancer Therapeutics Response Portal
CGGA	Chinese Glioma Genome Atlas
GSCA	Gene Set Cancer Analysis
GDSC	Genomics of Drug Sensitivity in Cancer
GBM	Glioblastoma multiforme
GO	Gene Ontology
HRs	Hazard ratios
IDH	Isocitrate dehydrogenase
KM	Kaplan–Meier
KEGG	Kyoto Encyclopedia of Genes and Genomes
LGG	Low-grade glioma
LD	Lipid droplets
MF	Molecular functions
MAIT	Mucosal-associated invariant T cells
OS	Overall survival
PLIN	Perilipin
PFS	Progression-Free Survival
TCGA	The Cancer Genome Atlas
TIMER	Tumor Immune Estimation Resource
WHO	World Health Organization

## References

[1] Louis D.N., Perry A., Wesseling P., Brat D.J., Cree I.A., Figarella-Branger D., Hawkins C., Ng H.K., Pfister S.M., Reifenberger G. (2021). The 2021 WHO Classification of Tumors of the Central Nervous System: A summary. Neuro-Oncology.

[2] Wang Z., Dai Z., Zheng L., Xu B., Zhang H., Fan F., Zhang X., Liang X., Liu Z., Yang K. (2021). Ferroptosis Activation Scoring Model Assists in Chemotherapeutic Agents’ Selection and Mediates Cross-Talk With Immunocytes in Malignant Glioblastoma. Front. Immunol..

[3] Lu H., Xiao J., Ke C., Ni X., Xiu R., Tian Q., Pan H., Zou L., Wang F., Ma T. (2019). TOPK inhibits autophagy by phosphorylating ULK1 and promotes glioma resistance to TMZ. Cell Death Dis..

[4] Wang X., Han M., Chen S., Sun Y., Tan R., Huang B. (2022). The copper-associated protein STEAP2 correlated with glioma prognosis and immune infiltration. Front. Cell. Neurosci..

[5] Youssef G., Miller J.J. (2020). Lower Grade Gliomas. Curr. Neurol. Neurosci. Rep..

[6] Zhou J., Zhao J., Su C. (2021). Role of Aberrant Lipid Metabolism of Cancer Stem Cells in Cancer Progression. Curr. Cancer Drug Targets.

[7] Bartz R., Li W.-H., Venables B., Zehmer J.K., Roth M.R., Welti R., Anderson R.G.W., Liu P., Chapman K.D. (2007). Lipidomics reveals that adiposomes store ether lipids and mediate phospholipid traffic. J. Lipid Res..

[8] Bozza P.T., Viola J.P.B. (2010). Lipid droplets in inflammation and cancer. Prostaglandins Leukot. Essent. Fat. Acids.

[9] Den Brok M.H., Raaijmakers T.K., Collado-Camps E., Adema G.J. (2018). Lipid Droplets as Immune Modulators in Myeloid Cells. Trends Immunol..

[10] Scorletti E., Carr R.M. (2022). A new perspective on NAFLD: Focusing on lipid droplets. J. Hepatol..

[11] Bickel P.E., Tansey J.T., Welte M.A. (2009). PAT proteins, an ancient family of lipid droplet proteins that regulate cellular lipid stores. Biochim. Biophys. Acta-Mol. Cell Biol. Lipids.

[12] Tauchi-Sato K., Ozeki S., Houjou T., Taguchi R., Fujimoto T. (2002). The surface of lipid droplets is a phospholipid monolayer with a unique fatty acid composition. J. Biol. Chem..

[13] Zhang T., Liu J., Tong Q., Lin L. (2020). SIRT3 Acts as a Positive Autophagy Regulator to Promote Lipid Mobilization in Adipocytes via Activating AMPK. Int. J. Mol. Sci..

[14] Zhang P., Meng L., Song L., Du J., Du S., Cui W., Liu C., Li F. (2018). Roles of Perilipins in Diseases and Cancers. Curr. Genom..

[15] Gandotra S., Le Dour C., Bottomley W., Cervera P., Giral P., Reznik Y., Charpentier G., Auclair M., Delepine M., Barroso I. (2011). Perilipin Deficiency and Autosomal Dominant Partial Lipodystrophy. N. Engl. J. Med..

[16] Langlois D., Forcheron F., Li J.-Y., Del Carmine P., Neggazi S., Beylot M. (2011). Increased Atherosclerosis in Mice Deficient in Perilipin1. Lipids Health Dis..

[17] Carr R.M., Peralta G., Yin X., Ahima R.S. (2014). Absence of Perilipin 2 Prevents Hepatic Steatosis, Glucose Intolerance and Ceramide Accumulation in Alcohol-Fed Mice. PLoS ONE.

[18] Shinozaki A., Nagao T., Endo H., Kato N., Hirokawa M., Mizobuchi K., Komatsu M., Igarashi T., Yokoyama M., Masuda S. (2008). Sebaceous epithelial-myoepithelial carcinoma of the salivary gland: Clinicopathologic and immunohistochemical analysis of 6 cases of a new histologic variant. Am. J. Surg. Pathol..

[19] Straub B.K., Herpel E., Singer S., Zimbelmann R., Breuhahn K., Macher-Goeppinger S., Warth A., Lehmann-Koch J., Longerich T., Heid H. (2010). Lipid droplet-associated PAT-proteins show frequent and differential expression in neoplastic steatogenesis. Mod. Pathol..

[20] Qu L.-W., Zhou B., Wang G.-Z., Chen Y., Zhou G.-B. (2017). Genomic variations in paired normal controls for lung adenocarcinomas. Oncotarget.

[21] Zhang X., Su L., Sun K. (2021). Expression status and prognostic value of the perilipin family of genes in breast cancer. Am. J. Transl. Res..

[22] Verhaak R.G.W., Hoadley K.A., Purdom E., Wang V., Qi Y., Wilkerson M.D., Miller C.R., Ding L., Golub T., Mesirov J.P. (2010). Integrated Genomic Analysis Identifies Clinically Relevant Subtypes of Glioblastoma Characterized by Abnormalities in PDGFRA, IDH1, EGFR, and NF1. Cancer Cell.

[23] Li T., Fu J., Zeng Z., Cohen D., Li J., Chen Q., Li B., Liu X.S. (2020). TIMER2.0 for analysis of tumor-infiltrating immune cells. Nucleic Acids Res..

[24] Chandrashekar D.S., Karthikeyan S.K., Korla P.K., Patel H., Shovon A.R., Athar M., Netto G.J., Qin Z.S., Kumar S., Manne U. (2022). UALCAN: An update to the integrated cancer data analysis platform. Neoplasia.

[25] Chandrashekar D.S., Bashel B., Balasubramanya S.A.H., Creighton C.J., Ponce-Rodriguez I., Chakravarthi B.V.S.K., Varambally S. (2017). UALCAN: A Portal for Facilitating Tumor Subgroup Gene Expression and Survival Analyses. Neoplasia.

[26] Edwards N.J., Oberti M., Thangudu R.R., Cai S., McGarvey P.B., Jacob S., Madhavan S., Ketchum K.A. (2015). The CPTAC Data Portal: A Resource for Cancer Proteomics Research. J. Proteome Res..

[27] Ponten F., Jirstrom K., Uhlen M. (2008). The Human Protein Atlas—A tool for pathology. J. Pathol..

[28] Bowman R.L., Wang Q., Carro A., Verhaak R.G.W., Squatrito M. (2017). GlioVis data portal for visualization and analysis of brain tumor expression datasets. Neuro-Oncology.

[29] Li X., Kang K., Peng Y., Shen L., Shen L., Zhou Y. (2022). Comprehensive analysis of the expression profile and clinical implications of regulator of chromosome condensation 2 in pan-cancers. Aging.

[30] Cerami E., Gao J., Dogrusoz U., Gross B.E., Sumer S.O., Aksoy B.A., Jacobsen A., Byrne C.J., Heuer M.L., Larsson E. (2012). The cBio Cancer Genomics Portal: An Open Platform for Exploring Multidimensional Cancer Genomics Data. Cancer Discov..

[31] Gao J., Aksoy B.A., Dogrusoz U., Dresdner G., Gross B., Sumer S.O., Sun Y., Jacobsen A., Sinha R., Larsson E. (2013). Integrative Analysis of Complex Cancer Genomics and Clinical Profiles Using the cBioPortal. Sci. Signal..

[32] Hu J., Yu A., Othmane B., Qiu D., Li H., Li C., Liu P., Ren W., Chen M., Gong G. (2021). Siglec15 shapes a non-inflamed tumor microenvironment and predicts the molecular subtype in bladder cancer. Theranostics.

[33] Hu J., Qiu D., Yu A., Hu J., Deng H., Li H., Yi Z., Chen J., Zu X. (2021). YTHDF1 Is a Potential Pan-Cancer Biomarker for Prognosis and Immunotherapy. Front. Oncol..

[34] Vasaikar S.V., Straub P., Wang J., Zhang B. (2018). LinkedOmics: Analyzing multi-omics data within and across 32 cancer types. Nucleic Acids Res..

[35] Zhou Y., Zhou B., Pache L., Chang M., Khodabakhshi A.H., Tanaseichuk O., Benner C., Chanda S.K. (2019). Metascape provides a biologist-oriented resource for the analysis of systems-level datasets. Nat. Commun..

[36] Reinhold W.C., Sunshine M., Liu H., Varma S., Kohn K.W., Morris J., Doroshow J., Pommier Y. (2012). CellMiner: A Web-Based Suite of Genomic and Pharmacologic Tools to Explore Transcript and Drug Patterns in the NCI-60 Cell Line Set. Cancer Res..

[37] Kuniyoshi S., Miki Y., Sasaki A., Iwabuchi E., Ono K., Onodera Y., Hirakawa H., Ishida T., Yoshimi N., Sasano H. (2019). The significance of lipid accumulation in breast carcinoma cells through perilipin 2 and its clinicopathological significance. Pathol. Int..

[38] Qiu B., Ackerman D., Sanchez D.J., Li B., Ochocki J.D., Grazioli A., Bobrovnikova-Marjon E., Diehl J.A., Keith B., Simon M.C. (2015). HIF2 alpha-Dependent Lipid Storage Promotes Endoplasmic Reticulum Homeostasis in Clear-Cell Renal Cell Carcinoma. Cancer Discov..

[39] Zhou C., Wang M., Zhou L., Zhang Y., Liu W., Qin W., He R., Lu Y., Wang Y., Chen X.-Z. (2016). Prognostic significance of PLIN1 expression in human breast cancer. Oncotarget.

[40] Greenberg A.S., Egan J.J., Wek S.A., Garty N.B., Blanchette-Mackie E.J., Londos C. (1991). Perilipin, a major hormonally regulated adipocyte-specific phosphoprotein associated with the periphery of lipid storage droplets. J. Biol. Chem..

[41] Wolins N.E., Quaynor B.K., Skinner J.R., Schoenfish M.J., Tzekov A., Bickel P.E. (2005). S3-12, Adipophilin, and TIP47 package lipid in adipocytes. J. Biol. Chem..

[42] Bensaad K., Favaro E., Lewis C.A., Peck B., Lord S., Collins J.M., Pinnick K.E., Wigfield S., Buffa F.M., Li J.-L. (2014). Fatty Acid Uptake and Lipid Storage Induced by HIF-1 alpha Contribute to Cell Growth and Survival after Hypoxia-Reoxygenation. Cell Rep..

[43] Zhang X.-D., Li W., Zhang N., Hou Y.-L., Niu Z.-Q., Zhong Y.-J., Zhang Y.-P., Yang S.-Y. (2014). Identification of adipophilin as a potential diagnostic tumor marker for lung adenocarcinoma. Int. J. Clin. Exp. Med..

[44] Szigeti A., Minik O., Hocsak E., Pozsgai E., Boronkai A., Farkas R., Balint A., Bodis J., Sumegi B., Bellyei S. (2009). Preliminary Study of TIP47 as a Possible New Biomarker of Cervical Dysplasia and Invasive Carcinoma. Anticancer Res..

[45] Zhou L., Song Z., Hu J., Liu L., Hou Y., Zhang X., Yang X., Chen K. (2021). ACSS3 represses prostate cancer progression through downregulating lipid droplet-associated protein PLIN3. Theranostics.

[46] Brat D.J., Verhaak R.G.W., Aldape K.D., Yung W.K.A., Salama S.R., Cooper L.A.D., Rheinbay E., Miller C.R., Vitucci M., Morozova O. (2015). Comprehensive, Integrative Genomic Analysis of Diffuse Lower-Grade Gliomas. N. Engl. J. Med..

[47] Chen L., Xiong Z., Zhao H., Teng C., Liu H., Huang Q., Wanggou S., Li X. (2022). Identification of the novel prognostic biomarker, MLLT11, reveals its relationship with immune checkpoint markers in glioma. Front. Oncol..

[48] Satish L., Krill-Burger J.M., Gallo P.H., Etages S.D., Liu F., Philips B.J., Ravuri S., Marra K.G., LaFramboise W.A., Kathju S. (2015). Expression analysis of human adipose-derived stem cells during in vitro differentiation to an adipocyte lineage. BMC Med. Genom..

[49] Zhang Q., Zhang P., Li B., Dang H., Jiang J., Meng L., Zhang H., Zhang Y., Wang X., Li Q. (2020). The Expression of Perilipin Family Proteins can be used as Diagnostic Markers of Liposarcoma and to Differentiate Subtypes. J. Cancer.

[50] Kuramoto K., Okamura T., Yamaguchi T., Nakamura T.Y., Wakabayashi S., Morinaga H., Nomura M., Yanase T., Otsu K., Usuda N. (2012). Perilipin 5, a Lipid Droplet-binding Protein, Protects Heart from Oxidative Burden by Sequestering Fatty Acid from Excessive Oxidation. J. Biol. Chem..

[51] Asimakopoulou A., Vucur M., Luedde T., Schneiders S., Kalampoka S., Weiss T.S., Weiskirchen R. (2019). Perilipin 5 and Lipocalin 2 Expression in Hepatocellular Carcinoma. Cancers.

[52] Hashani M., Witzel H.R., Pawella L.M., Lehmann-Koch J., Schumacher J., Mechtersheimer G., Schnoelzer M., Schirmacher P., Roth W., Straub B.K. (2018). Widespread expression of perilipin 5 in normal human tissues and in diseases is restricted to distinct lipid droplet subpopulations. Cell Tissue Res..

[53] Ye J., Liu H., Xu Z.-L., Zheng L., Liu R.-Y. (2019). Identification of a multidimensional transcriptome prognostic signature for lung adenocarcinoma. J. Clin. Lab. Anal..

[54] Zhou Y., Li X., Long G., Tao Y., Zhou L., Tang J. (2022). Identification and validation of a tyrosine metabolism-related prognostic prediction model and characterization of the tumor microenvironment infiltration in hepatocellular carcinoma. Front. Immunol..

[55] Gerard C.L., Delyon J., Wicky A., Homicsko K., Cuendet M.A., Michielin O. (2021). Turning tumors from cold to inflamed to improve immunotherapy response. Cancer Treat. Rev..

[56] Hu K., Yao L., Yan Y., Zhou L., Li J. (2021). Comprehensive Analysis of YTH Domain Family in Lung Adenocarcinoma: Expression Profile, Association with Prognostic Value, and Immune Infiltration. Dis. Markers.

[57] Kim J., Bae J.-S. (2016). Tumor-Associated Macrophages and Neutrophils in Tumor Microenvironment. Mediat. Inflamm..

[58] Qiu Y., Chen T., Hu R., Zhu R., Li C., Ruan Y., Xie X., Li Y. (2021). Next frontier in tumor immunotherapy: Macrophage-mediated immune evasion. Biomark. Res..

[59] Mishra A.K., Banday S., Bharadwaj R., Ali A., Rashid R., Kulshreshtha A., Malonia S.K. (2022). Macrophages as a Potential Immunotherapeutic Target in Solid Cancers. Vaccines.

[60] Mantovani A., Allavena P., Marchesi F., Garlanda C. (2022). Macrophages as tools and targets in cancer therapy. Nat. Rev. Drug Discov..

