# Supplementary material for: Integrative Analysis of the Predictive Value of Perilipin Family on Clinical Significance, Prognosis and Immunotherapy of Glioma
Source: Biomedicines. 2023 Mar 24;11(4):1009. doi: 10.3390/biomedicines11041009 (PMC10136080; doi:10.3390/biomedicines11041009)
Supplement: Supplementary file 1 [file biomedicines-11-01009-s001.zip › biomedicines-2199361-supplementary.pdf]

Supplementary Materials

Integrative Analysis of the Predictive Value of Perilipin Family on Clinical Significance, Prognosis and Immunotherapy of Glioma

Xuanxuan Li [1,2,†], Kuo Kang [3,4,†], Lin Shen [1], Liangfang Shen [1] and Yangying Zhou [1,2,*]

1 Department of Oncology, Xiangya Hospital, Central South University, Changsha 410008, China
2 National Clinical Research Center for Geriatric Disorders, Xiangya Hospital, Central South University, Changsha 410008, China
3 Department of General Surgery, Xiangya Hospital, Central South University, Changsha 410008, China
4 Hunan Key Laboratory of Precise Diagnosis and Treatment of Gastrointestinal Tumor, Xiangya Hospital, Central South University, Changsha 410008, China
* Correspondence: 22021144@csu.edu.cn
† These authors contributed equally to this work.

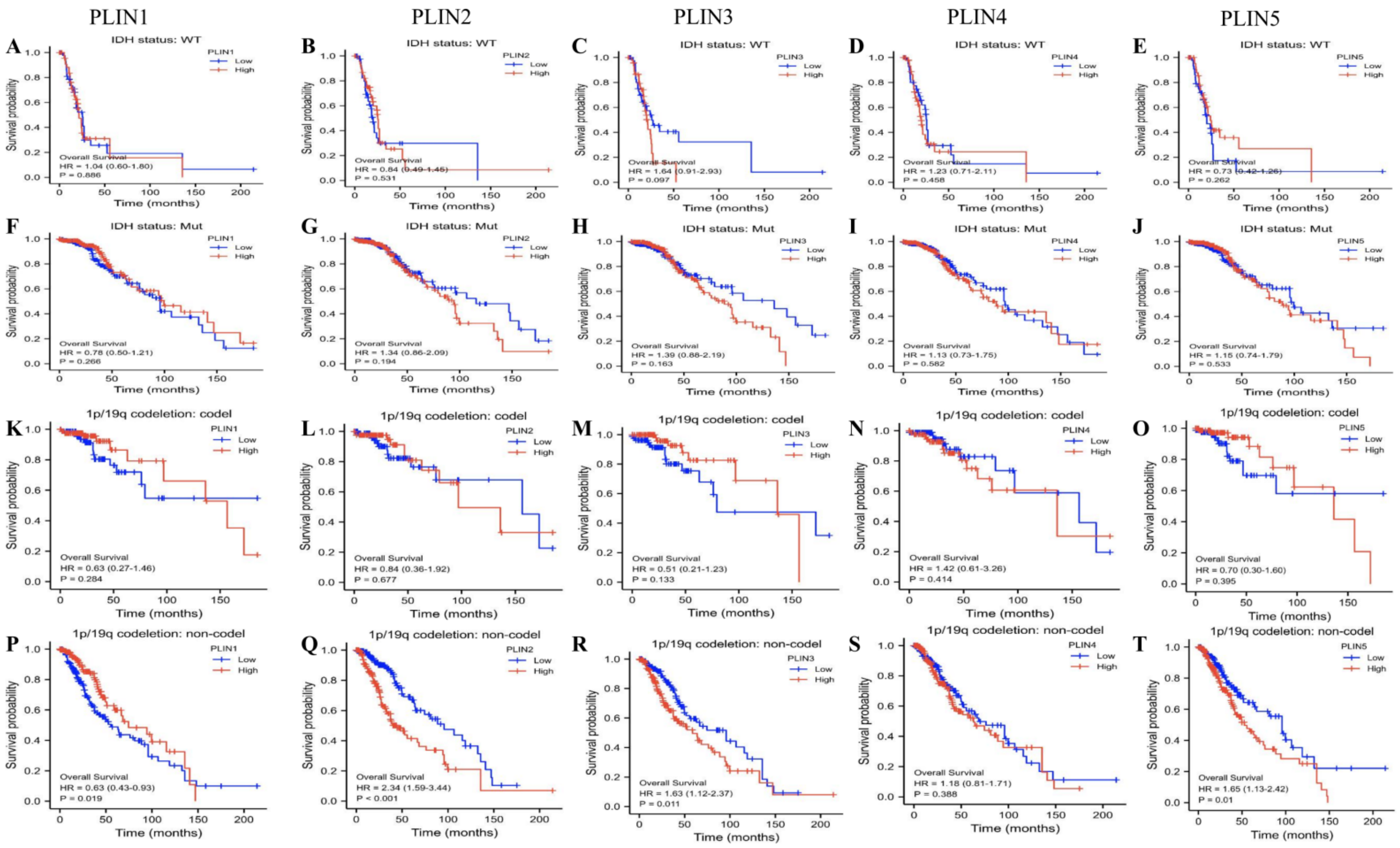

Figure S1. Prognostic analysis of *PLINs* family in different subgroups of LGG. (A-J) Overall survival analysis of *PLIN1-5* mRNA expression in LGG IDH mutant and wild type.**(K-T)** Overall survival analysis of *PLIN1-5* mRNA expression in LGG 1p/19q coding and non-coding.

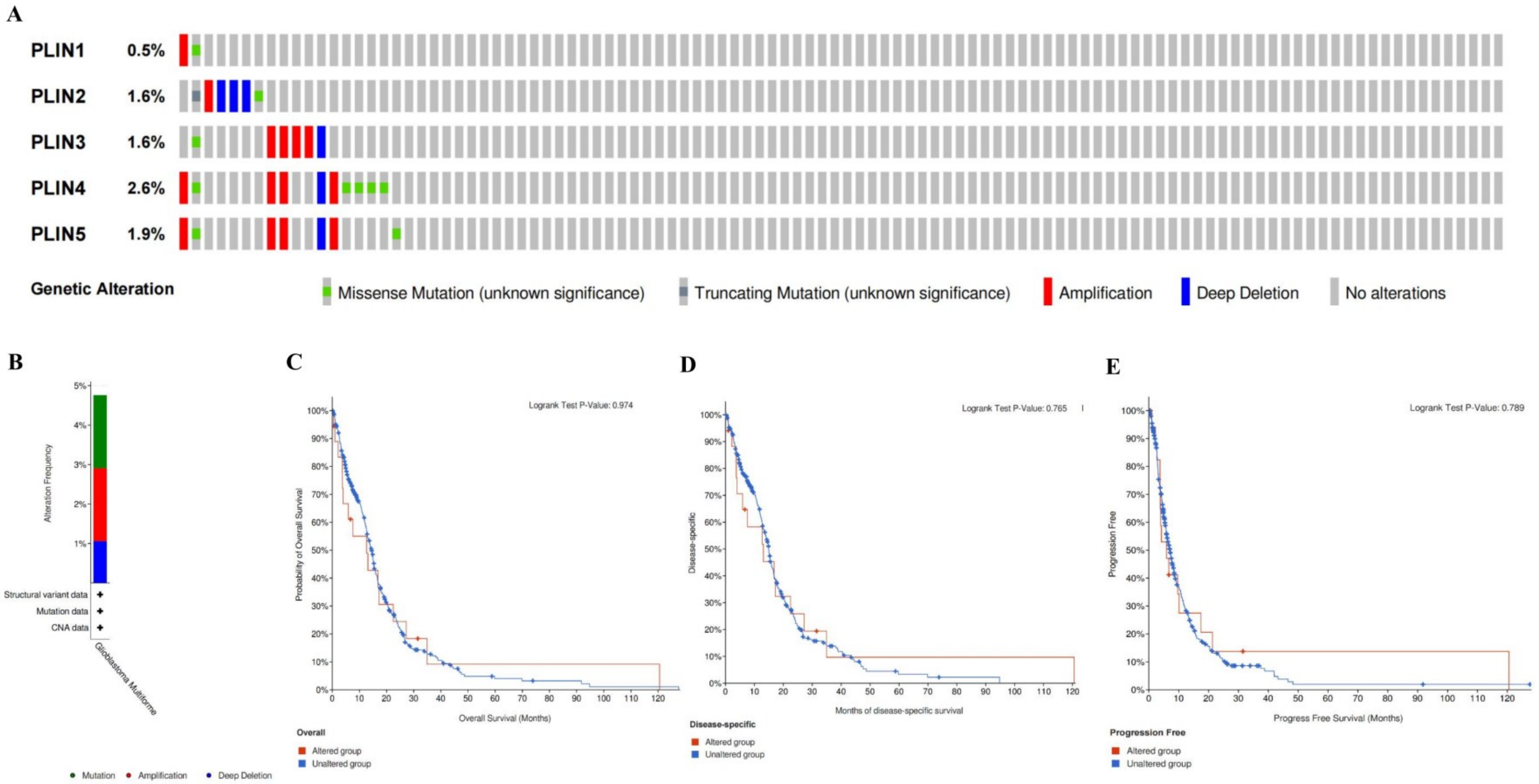

Figure S2. Genetic alterations of the *PLINs* family in GBM.(A-B) Frequency and type of genetic alterations of *PLINs* in GBM. **(C-E)** Prognostic analysis of genetically altered and non-altered groups of *PLINs* families in GBM.

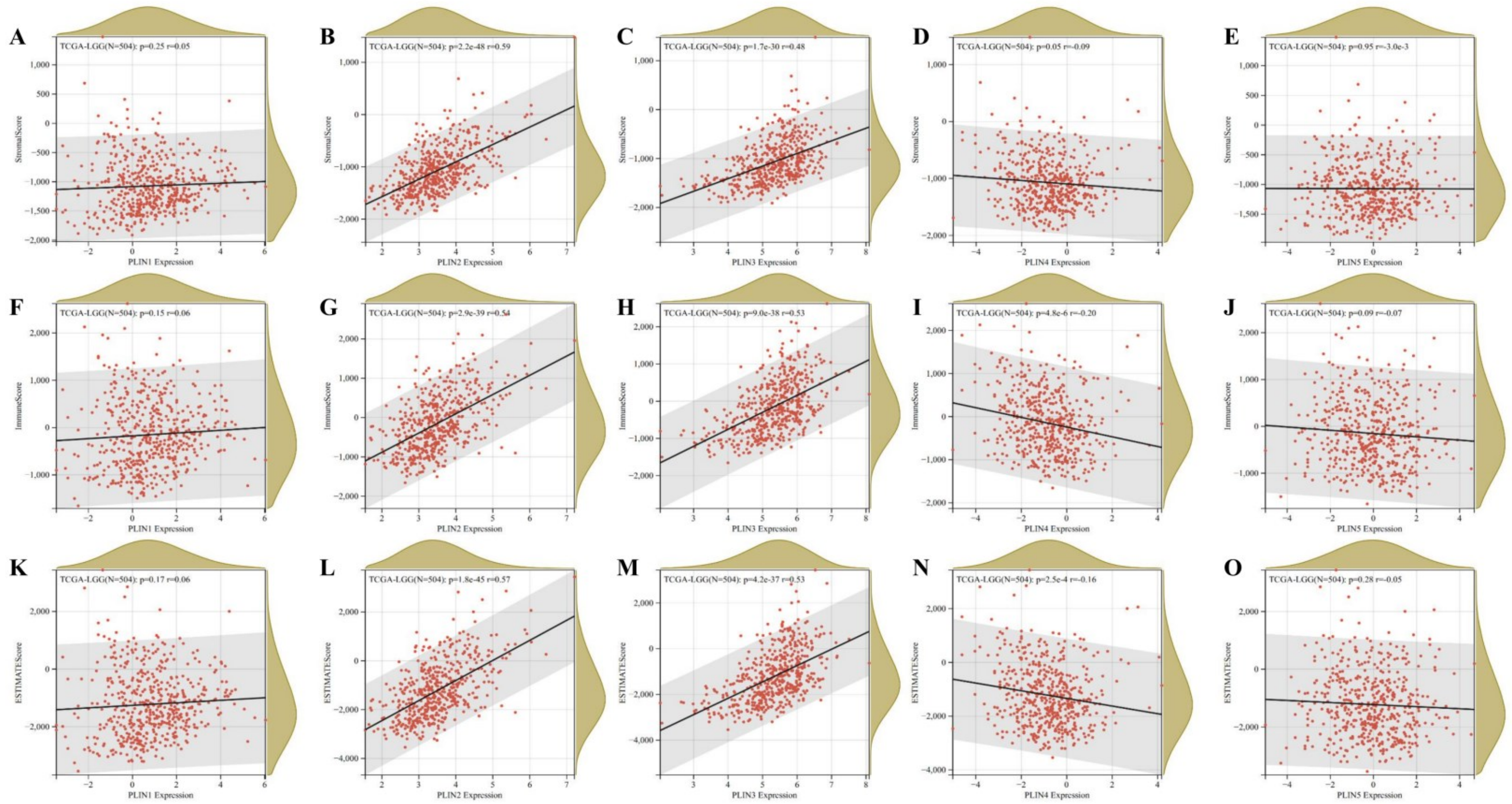

Figure S3. Immune infiltration score of *PLINs* family in LGG.(A) Correlation of *PLIN1-5* expression with stromal scores in LGG. **(B)** Correlation of *PLIN1-5* expression with immune scores in LGG. **(C)** Correlation of *PLIN1-5* expression with ESTIMATE scores in LGG.

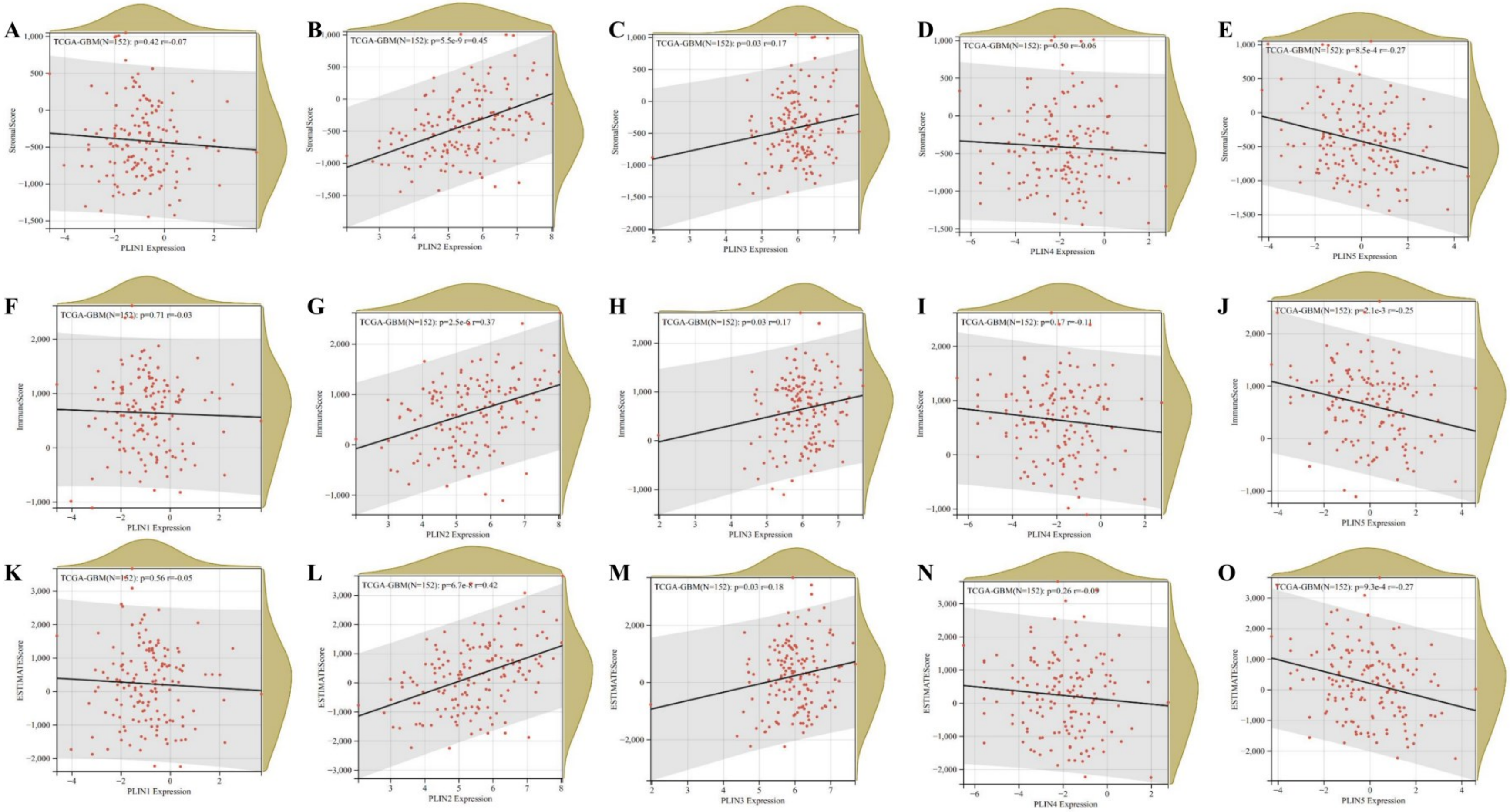

Figure S4. Immune infiltration score of *PLINs* family in GBM.(A) Correlation of *PLIN1-5* expression with stromal scores in GBM. **(B)** Correlation of *PLIN1-5* expression with immune scores in GBM. **(C)** Correlation of *PLIN1-5* expression with ESTIMATE scores in GBM.

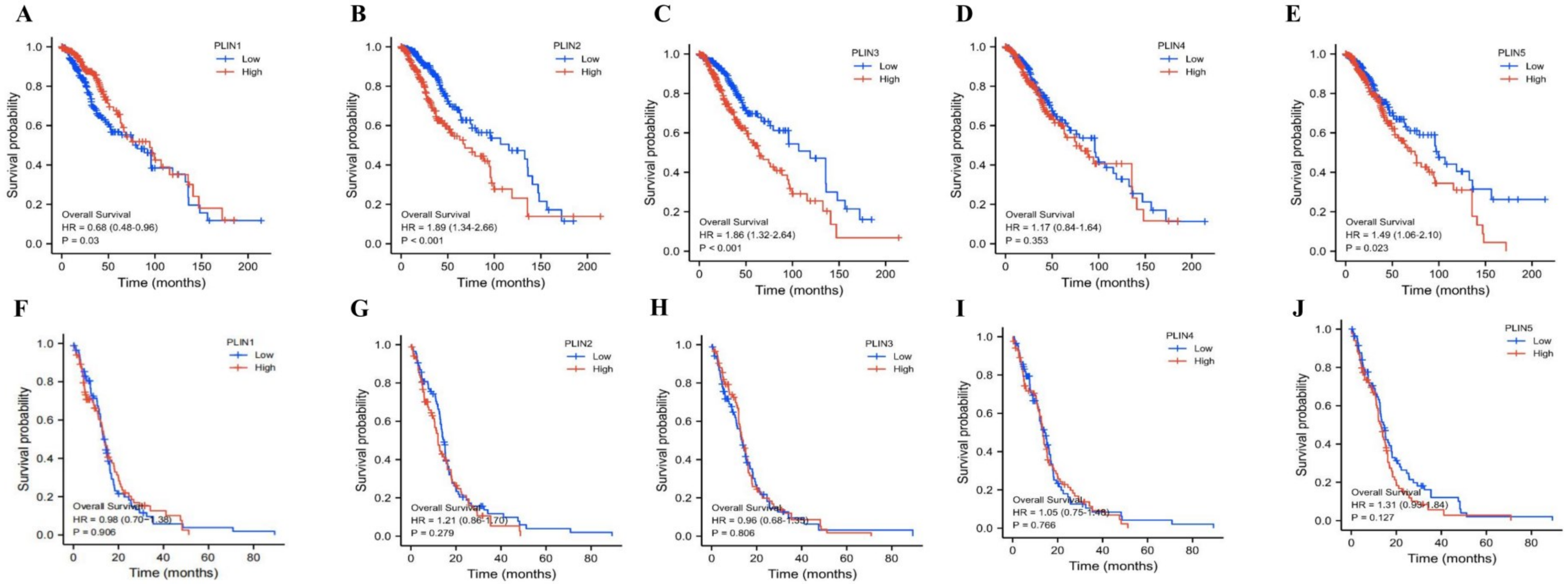

Figure S5. Correlations between *PLINs* expression and drug sensitivity from Cellminer database. (A-C) The expression of *PLIN1* was negatively correlated with the drug sensitivity of Trametinib, AEE-788 and Adavosertib. **(D-E)** The relationship between *PLIN2* and drug reactivity of Dabrafenib and Selumetinib. **(F)** The correlation between *PLIN3* and drug reactivity of Telatinib. (G) The correlation between *PLIN4* and drug reactivity of Trametinib. (H) The correlation between *PLIN5* and drug reactivity of Linsitinib.

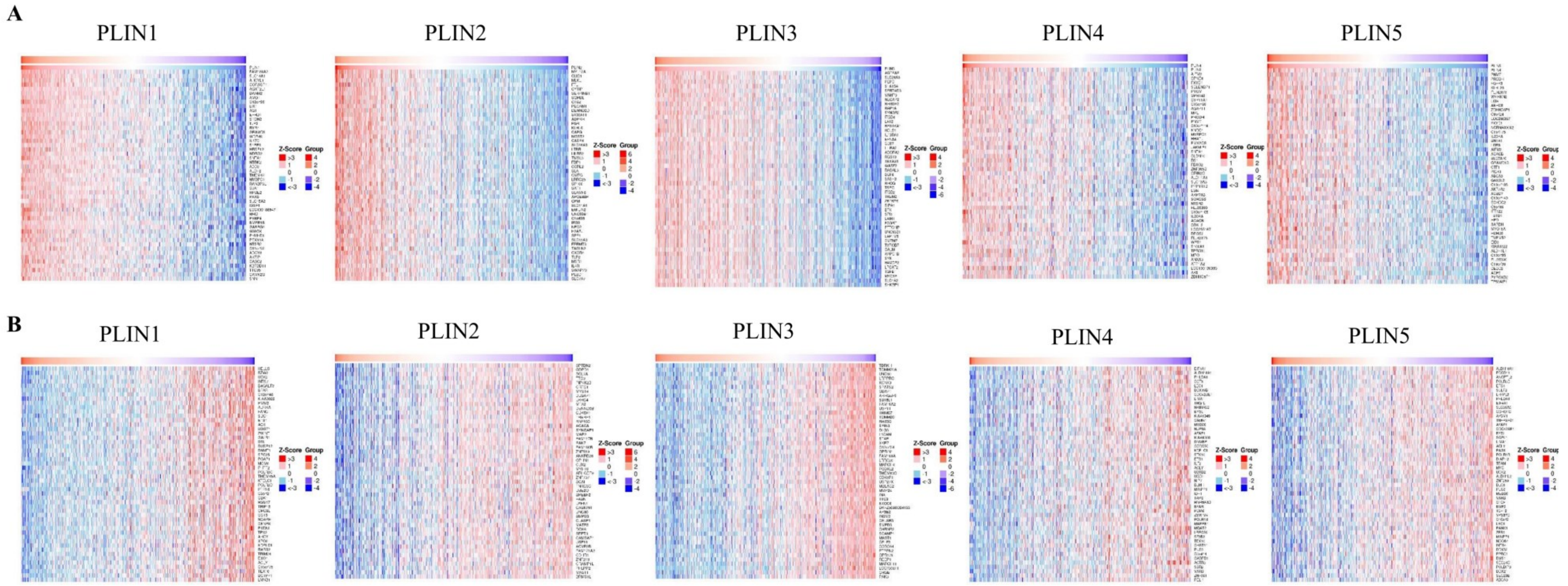

Figure S6. Heat map of *PLINs* family gene-related genes in LGG patients. (A) Top 50 genes positively associated with *PLINs* family genes. **(B)** Top 50 genes negatively associated with *PLINs* family genes.

Table S1. Clinicopathological characteristics of patients from TCGA cohort.

Characteristics	Category	Number of cases	(%)
**Age, median (IQR)**	45 (34, 59)		
**Gender**			
	Male	401	57.4
	Female	298	42.6
**Histological type**			
	Astrocytoma	196	28.0
	Oligoastrocytoma	135	19.3
	Oligodendroglioma	200	28.6
	Glioblastoma	168	24
**WHO grade**			
	G2	224	35.2
	G3	245	38.5
	G4	168	26.4

Table S2. Clinicopathological characteristics of patients from CGGA cohort.

Characteristics	Category	Number of cases	(%)
**Age, median (IQR)**	42 (35, 51)		
**Gender**			
	Male	601	59.0
	Female	417	41.0
**Histological type**			
	Astrocytoma	175	17.2
	Oligoastrocytoma	9	0.9
	Oligodendroglioma	112	11.0
	Anaplastic oligodendroglioma	94	9.2
	Anaplastic oligoastrocytoma	21	2.1
	Anaplastic astrocytoma	214	21.0
	Glioblastoma	388	38.1
	Unknown	5	0.5
**WHO grade**			
	G2	291	28.6
	G3	334	32.8
	G4	388	38.1
